# Interleukin-17: A Promoter in Colorectal Cancer Progression

**DOI:** 10.1155/2013/436307

**Published:** 2013-12-07

**Authors:** Dang Wu, Pin Wu, Qi Huang, Yang Liu, Jun Ye, Jian Huang

**Affiliations:** Cancer Institute (Key Laboratory of Cancer Prevention and Intervention, Key Laboratory of Molecular Biology in Medical Sciences, China National Ministry of Education), Second Affiliated Hospital, Zhejiang University School of Medicine, 88 Jiefang Road, Hangzhou, Zhejiang 310009, China

## Abstract

It is widely accepted that chronic inflammation plays an active role in cancer. Inflammatory immunocytes and related cytokines in the tumor microenvironment are supposed to be a “double-edged sword” in colorectal cancer (CRC) initiation and progression. Interleukin-17 (IL-17), a pleiotropic proinflammatory cytokine, can promote cancer-elicited inflammation and prevent cancer cells from immune surveillance. Despite controversy, IL-17 is generally considered to be a promoter in CRC progression. In this review, we devote to summarize the current progress regarding the role of IL-17 in tumor initiation and progression, as well as the prognostic value in CRC.

## 1. Introduction

Human colorectal cancer (CRC) is an important contributor to cancer mortality and morbidity in developed countries [1]. It is generally accepted that most solid tumors including CRC are linked to chronic inflammation [2]. Due to various inflammatory cells and cytokines in tumor microenvironment, these tumors have been referred to as ‘‘wounds that do not heal” [3]. Accumulating evidence has shown that the functional disturbance of the immune system is directly associated with tumor stages and clinical prognosis of patients [4]. However, the interaction between immune cells, inflammatory cytokines, and cancer evolution is still largely unknown. Plentiful studies have demonstrated that innate and adaptive immunity play a critical role in the initiation and progression of CRC [5]. Recently, several inflammatory cytokines have been shown to promote CRC progression [6].

IL-17 (IL-17A), initially termed as cytotoxic T-lymphocyte-associated antigen (CTLA)-8, is the founding member of IL-17 cytokine family consisting of six homologous proteins (from IL-17A to IL-17F) [7, 8]. A large body of evidence suggests that IL-17 is an essential proinflammatory cytokine due to inducing a mass of cytokines and chemokines secretion by distinct cell types, such as mesenchymal cells and myeloid cells, which recruit monocytes and neutrophils into the site of inflammation [9]. Moreover, IL-17 promotes the expression of antimicrobial peptides from epithelial cells and facilitates host defense against infections [10, 11]. This evidence indicates that IL-17 is an important inflammatory cytokine which links innate and adaptive immunity. Besides, IL-17 has been demonstrated to play an active role in allergy, autoimmune diseases, allograft transplantation, and cancer [12–15]. Recently, several studies have shown that IL-17 has either a protumor or antitumor role in different cancer models [16]. But in CRC, the majority of studies consider that IL-17 acts as a promoter in tumor initiation and progression. Particularly, the ablation of IL-17A can inhibit the progression of spontaneous intestinal tumorigenesis in Apc^Min/+^ mice [17]. In this review, we devote to summarize the progress of the current study about the potential role of IL-17 in CRC initiation and progression, as well as its predictive role in clinical prognosis.

## 2. The Regulation of IL-17 Secretion

**Figure 1:**
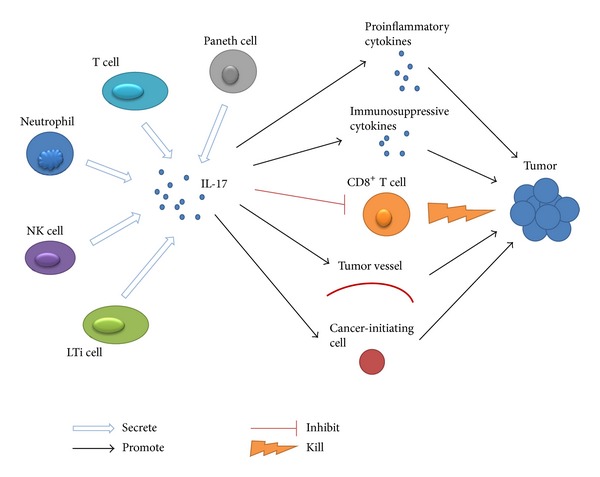
This is an illustration about the protumor activity of IL-17 in CRC microenvironment. Blue colored arrow shows cells producing IL-17. Black arrow shows the latter stimulated by the former. T-shaped arrow shows process inhibited by IL-17 and lightning arrow shows attack on tumor cells.

IL-17 is an inflammatory cytokine produced by a wide variety of leukocytes, as illustrated in Figure 1, including T cells, natural killer cells (NK cells), lymphoid tissue inducer-like cells (LTi-like cells), and neutrophils [18]. Among these cells, IL-17 is reported to be predominantly produced by activated CD4^+^ T cells (Th17 cells). It is generally accepted that Th17 cells are induced from naive CD4^+^ T cells by IL-6, IL-1*β*, TGF-*β*, and IL-23, which upregulate the expression of retinoic acid receptor-related orphan receptor-*γ*t (ROR*γ*t) via activation of signal transducer and activator of transcription-3 (Stat3) and interferon regulatory factor 4 (IRF4) [19, 20]. Other transcriptional factors such as RORa, basic leucine zipper transcription factor (Batf), Runx1, and aryl hydrocarbon receptor (AHR) can also induce Th17 cell polarization when coordinated with ROR*γ*t [21]. Moreover, the regulation mechanism of IL-17 production in Th17 cells is affected by other inflammatory immunocytes and related cytokines. For instance, human inflammatory dendritic cells (infDCs), derived from monocytes, can stimulate autologous memory CD4^+^ T cells to produce IL-17 [22]. Recently, IL-27 is reported to inhibit IL-17 production in early stage of Th17 cells differentiation through PD-1-PD-L1 interaction [23]. However, the cells or related cytokines negatively regulating IL-17 production are largely unknown.

In addition to Th17 cells, IL-17 secretion can also be induced by IL-6, IL-1*β*, TGF-*β*, and IL-23 in other T cells such as CD8^+^ T cells (Tc17 cells), regulatory T cells (Treg17 cells), gamma delta T cells (*γδ*T17 cells), and invariant natural killer T cells (iNKT cells) [24–26]. Moreover, it has been reported that intestinal Paneth cells (Figure 1) are capable of producing IL-17 [27]. Based on these results, we conclude that IL-17 secretion is regulated by the cooperation of the inflammatory cells, cytokines, and antigens which coexist in the specific inflammatory microenvironment.

## 3. The Role and Mechanism of IL-17 in Cancer Promotion

IL-17 expression is elevated in several human tumors, such as ovarian cancer, cervical cancer, breast cancer, hepatocellular carcinoma, esophageal cancer, gastric cancer, and CRC [28–34]. But the underlying mechanism of IL-17 in tumor initiation and progression is not completely clear yet. Some researchers propose that IL-17 promotes tumor initiation and progression through suppressing antitumor immune response. For instance, CD8^+^ T cells are polarized towards an IL-17 secreting (Tc17) fate in the presence of both TGF-*β* and IL-6, resulting in losing their cytotoxic ability and promoting tumorigenesis [35]. In gastric cancer, activated monocytes promote the development of Tc17 cells via IL-6, IL-1*β*, and IL-23, resulting in the production of the chemokine CXCL12 by tumor cells, which promotes MDSCs-mediated immunosuppression [33]. Other investigators demonstrate that IL-17 can enhance tumor progression through angiogenesis. IL-17 induces fibroblasts and tumor cells to produce a variety of angiogenic factors, including PGE1, PGE2, VEGF, keratinocyte-derived chemokine (KC), and macrophage inflammatory protein-2 (MIP-2), which promotes angiogenesis in the tumor [36]. In breast cancer, the angiogenic factors CXCL8, MMP-2, MMP-9, and VEGF are induced by IL-17 and associated with poor prognosis [30]. Analogously, IL-17 has been demonstrated to selectively promote the secretion of an array of angiogenic chemokines from NSCLC, such as CXCL1, CXCL5, CXCL6, and CXCL8 [37]. The molecule mechanism involved in the protumor activity of IL-17 is considered to be mediated by inflammation-associated signaling pathways. Transfecting IL-17 into hepatocellular carcinoma cells significantly promotes neoangiogenesis, neutrophils recruitment, and tumor growth via AKT-dependent IL-6/JAK2/STAT3 signaling pathway *in vivo* [38]. IL-17 induces IL-6 production, which in turn activates Stat3 and promotes cancer cells survival [39]. Further studies on the molecule mechanism of IL-17 inducing tumor promotion are required in the future.

Besides, some studies suggest that IL-17 can inhibit tumor growth. In the tumor initiating model, IL-17 deficient mice are more susceptible to developing lung melanoma, and adoptive T-cell therapy with tumor-specific Th17 cells prevents tumor development by eliciting a remarkable activation of tumor-specific CD8^+^ T cells [40]. Study in hematopoietic cancer shows that IL-17 inhibits the tumor growth in a CTL-dependent manner [41]. Murine Meth-A fibrosarcoma cells transfected with the hIL-17 gene can promote CD4^+^ and CD8^+^ T-cells-mediated antitumor activity [42]. Interestingly, hIL-17-gene-transfected Chinese hamster ovary (CHO) cells show a significant decrease in metastatic potential to the lung by directly reducing the invasiveness of CHO cells and enhancing NK activity [43]. This evidence indicates that IL-17 may have partial antitumor effect through promoting immune response in the tumor initiation stage.

## 4. IL-17 as a Promoter in CRC Progression

In accordance with studies in other cancers, growing evidence has shown that IL-17 can also promote tumor progression in CRC. In intestinal tumor bearing model, the tumor size is significantly reduced in IL-17 gene-knockout mice compared with wide-type (WT) mice, and anti-IL-17A monoclonal antibody treatment results in decreased tumor size in the WT mice [44]. *In vitro*, IL-17 and TNF-*α* synergistically promote carcinogenesis by stimulating glycolysis and growth factor production by CRC cells [45]. Study focused on the intrinsic role of endogenous IL-17 in CRC has demonstrated that CD4^+^ T-cell-derived IL-17 promotes spontaneous intestinal tumorigenesis in Apc^Min/+^ mice, suggesting that IL-17 plays an important role in CRC initiation [17]. In colitis-associated cancer model, tumorigenesis and inflammatory cytokines including IL-6, IFN-*γ*, and TNF-*α* are markedly decreased in IL-17-deficient mice compared with WT mice, suggesting that IL-7 plays an pivotal role in promoting CRC initiation in colitis-associated cancer [46]. Enterotoxigenic bacteroides fragilis (ETBF), a human colonic commensal bacterium, can promote colonic tumorigenesis in APC mutant mice via Stat3 activation and Th17-cell polarization, and further blocking IL-17 or IL-23R can significantly reduce tumor formation *in vivo* [47]. It has been reported that epithelial barrier deterioration results in microbial pathogen invasion and microbial products release are driving IL-23/IL-17 axis activation and promoting tumor growth and progression in CPC-APC mice [48]. We have found that tumor-infiltrating *γδ*T17 cells induced by tumor-elicited inflammation can promote tumor progression via secretion of IL-17, IL-8, TNF-*α*, and GM-CSF to form an immunosuppressive microenvironment in human CRC (unpublished data). Similarly, another study has presented that a subset of Foxp3^+^ IL-17^+^ T cells in CRC tissue suppress the tumor-specific CD8^+^ T cells and attenuate the antitumor immune response [49]. Moreover, IL-17/G-CSF/Bv8 axis has been reported to promote VEGF-independent CRC tumor angiogenesis *in vivo* [50]. Recently, a study has suggested that CRC tissue-derived Foxp3^+^ IL-17^+^ cells have the capacity to induce cancer-initiating cells *in vitro* [51]. These studies regarding CRC promoting activities of IL-17 are highlighted in Table 1. Based on these findings, we propose that the protumor activity of IL-17 in CRC microenvironment may exert in several aspects: (1) promoting tumor-elicited inflammation which facilitates the proliferation and survival of malignant cells, (2) forming an immunosuppressive tumor microenvironment by chemoattracting immunosuppressive cells and cytokines, (3) suppressing cytotoxic cells-mediated immunosurveillance against tumor, (4) fostering tumor angiogenesis to promote tumor growth and metastasis, and (5) inducing cancer-initiating cells, which facilitates tumor malignant progression and escaping from host immune surveillance as shown in Figure 1.

Although major evidence considers IL-17 as a promoter in CRC progression, there is still controversy. For instance, Kryczek and colleagues have demonstrated that tumor growth is enhanced in subcutaneous transplanted model and lung metastases model in IL-17^−/−^  mice [53]. However, Ngiow et al. fail to reproduce the same results and they conclude that tumor growth has no difference between IL-17-deficient mice and control WT mice after 3 independent sources of MC38 cells inoculated subcutaneously [55]. Analogously, it has been reported that IL-17 promotes the expression of the tight junction protein-claudin in T84 cells via ERK MAPK activation in intestine [54]. Whereas, another study has shown that adenoma-linked barrier deterioration leads to microbial products invasion and triggers IL-23/IL-17-mediated tumor growth in CPC-APC mouse model [48]. In summary, despite the existing controversy presumably derived from the different models, most investigators appreciate IL-17 as a promoter in CRC progression. Table 1 summarizes studies with IL-17 which describe its possible antitumor role in CRC.

## 5. IL-17 as a Clinical Prognostic Indicator for Human CRC

Tumor progression is affected by the complicated interaction of tumor cells, stromal cells, immune cells, and related cytokines in tumor microenvironment. IL-17 produced by epithelial cells and immune cells plays an important role in CRC development. Increased IL-17 concentration is detected in serum of CRC patients compared with healthy donors, which is inversely correlated with p53 expression. Moreover, it is proposed that IL-17 may act as a valuable tumor marker in patients with CRC and that concomitant expression of p53 and VEGF may provide further information about tumor features [56]. Furthermore, elevated Th17 cells have been observed in more than 80% of human sporadic colon cancer tissues, indicating that IL-17 expression may be one of potential biomarkers for the future development of a new prognostic ‘‘test set” for sporadic CRC [34]. Analogously, IL-17 producing cells induced by microbial dysbiosis are increased in intestinal mucosa of CRC patients, indicating that IL-17 producing cells may be a promising sensitive prognostic indicator for CRC [57]. Confocal microscopic analysis of CRC tissues shows that IL-17 expression is associated with microvessel density. Univariate and multivariate analysis reveal that 5-year survival rate is 72.41% in the 26 cases with lower IL-17 expression and 38.08% in the 26 cases with higher IL-17 expression, proposing that IL-17 is an independent prognostic factor for overall survival and IL-17 producing cells may facilitate development of CRC by fostering angiogenesis via stimulation of VEGF production by cancer cells [52]. Interestingly, an early increase of IL-17 expression in the premalignant stage and its dynamic change in tumor microenvironment throughout the adenoma-carcinoma sequence is associated with the progression of adenomas toward CRC [58]. These clinical studies indicate that IL-17 plays a critical role in the human CRC progression, which deserves to be studied further.

Elevated IL-17 expression level in serum and tissue of CRC patients suggests that it may contribute to predicting cancer prognosis accompanied with an other existing panel of molecular prognosticator. However, more multicentric validated studies are required before considering IL-17 as an authentic prognosticator that can apply to clinical practices. Studies on IL-17 expression level and its significance associated with CRC are summarized in Table 2.

## 6. Concluding Remarks

In conclusion, accumulative evidence reveals that IL-17 can promote tumor initiation and progression in most cancers. Due to the complexity of tumor microenvironment contents, the underlying mechanism of IL-17 in CRC initiation and progression is still uncovered. Recently, our group has demonstrated that IL-17 promotes polymorphonuclear myeloid-derived suppressor cells (PMN-MDSCs) mediated immunosuppression in human CRC progression (unpublished data). Thus, some attempts for cancer immunotherapy targeting IL-17, such as neutralizing antibody, and approaches of decreasing IL-17 production or blocking the downstream signaling pathways would come true with the deepening of IL-17-related research in the future. On the other hand, because IL-17 may serve as a promising biomarker with certain prognostic significance and potential clinical application prospect, further studies that focus on IL-17 regulation mechanism and the interaction between IL-17, cancer cells, mesenchymal cells, and immune cells in tumor microenvironment are expected to shed more light both on the exact biological function of IL-17 in CRC development and the clinical applications of IL-17-targeted treatment.

## Figures and Tables

**Table 1:** Role of IL-17 in CRC.

Species	Mediators	Findings	References
Tumor promoting role			
Human	VEGF	IL-17 induces both CRC cell lines and primary cancer cells to produce VEGF.	[52]

Human	HIF-1*α* and c-myc	IL-17 and TNF*α* cooperatively stimulate glycolysis in CRC cells via induction of HIF-1*α* and c-myc expression.	[45]

Mouse	Stat 3	IL-23/IL-17 signaling activated by microbial products promotes STAT3 phosphorylation in CRC epithelial cells.	[48]

Mouse	VEGF, KC, and PGE2	IL-17 promotes angiogenesis via induction of a variety of proangiogenic factors secretion from fibroblasts and tumors.	[36]

Mouse	IL-6, IL-23, and IL-1*β*; KC and Cox-2; CD4 T cells	IL-6, IL-23, IL-1*β*, KC, and Cox-2 are decreased and function of CD4 T cells alters in Apc^Min/+^ mice, resulting in abrogating spontaneous intestinal tumorigenesis.	[17]

Mouse	IL-6, STAT3, and TNF-*α*; cyclin D1, cyclin-dependent kinase 2, and cyclin E	IL-17A knockout decreases IL-6, STAT3, TNF-*α*, cyclin-D1, cyclin-dependent kinase 2, and cyclin E and inhibits CAC tumorigenesis.	[46]

Mouse	G-CSF, VEGF, and Bv8 NF-*κ*B and ERK signaling	IL-17 induces the expression of G-CSF through NF-*κ*B and ERK signaling, enhancing proangiogenic function via VEGF and Bv98 and promoting tumor growth.	[50]

Tumor inhibiting activity			
Mouse	IFN-*γ* NK cells and T cells	IL-17-deficient decreases IFN-*γ* ^+^ NK and tumor-specific IFN-*γ* ^+^ T cells and promotes tumor growth and lung metastasis.	[53]

Human	Claudin ERK MAPK pathway	IL-17 enhances the development of the tight junctional barrier mediated by claudin of T84-cell monolayers via ERK MAPK pathway in intestine.	[54]

**Table 2:** Expression of IL-17 in CRC patients.

Sample type	Sample size	Findings	References
Serum and tissue	74 CRC tissues and paired normal mucosa, 61 CRC serum samples, and 78 healthy controls	No significant difference is observed.	[59]

Serum and tissue	59 CRC tissues, 40 CRC serum samples, and 37 healthy controls	IL-17 acts as a valuable tumor marker in CRC patients.	[56]

Tissue	12 CRC patients	Hypoxia induce the expression of IL-17 in Foxp3^+^ Tregs, which drive cancer cells to be cancer-initiating cells.	[51]

Tissue	125 CRC tissues and 3 normal tissues	IL-17 gene expression level is higher in tumor tissues compared to normal mucosa.	[60]

Tissue	52 CRC patients	IL-17 expression is negatively correlated with OS of CRC patients.	[52]

Tissue	22 CRC patients	Tumor-infiltrating Foxp3^+^ IL-17^+ ^T cells suppress tumor-specificCD8^+^T cells response via IL-17.	[49]

Serum and tissue	9 ulcerative colitis-associated CRC tissues	IL-17^+ ^Foxp3^+ ^CD4^+ ^T cells are selectively accumulated in the colitis-associated CRC niche.	[61]

Tissue	50 colorectal adenomas tissues, 50 CRC tissues, and 15 healthy controls	IL-17 level is associated with the severity of dysplastic degree.	[58]

Tissue	23 CRC patients	Tumor-infiltrating TH17 cells and Bv8-expressing neutrophils are associated with poor outcome in CRC.	[50]
